# Acceptance, Barriers, and Future Preferences of Mobile Health Among Patients Receiving Trauma and Orthopedic Surgical Care: Paper-Based Survey in a Prospective Multicenter Study

**DOI:** 10.2196/23784

**Published:** 2021-04-21

**Authors:** Felix Reinecke, Florian Dittrich, Marcel Dudda, Andreas Stang, Christina Polan, Roman Müller, Paula Beck, Max Daniel Kauther

**Affiliations:** 1 Department of Trauma, Hand and Reconstructive Surgery University Medicine Essen Essen Germany; 2 Department of Orthopedics and Orthopedic Surgery Saarland University Medical Center and Saarland University Faculty of Medicine Homburg Germany; 3 Joint Centre Bergischland Sana Fabricius Clinic Remscheid Remscheid Germany; 4 Institute of Medical Informatics, Biometry and Epidemiology University Hospital Essen Essen Germany

**Keywords:** smartphone, mHealth, app, orthopedics, trauma surgery

## Abstract

**Background:**

Smartphones have become an essential part of everyday life and it is undeniable that apps offer enormous opportunities for dealing with future challenges in public health. Nevertheless, the exact patient requirements for medical apps in the field of orthopedic and trauma surgery are currently unknown.

**Objective:**

The aim of this study was to define target groups, evaluate patient requirements, and the potential and pitfalls regarding medical apps specific for patients receiving orthopedic and trauma surgical care.

**Methods:**

A prospective multicenter study was conducted between August 2018 and December 2019 at a German trauma center and 3 trauma surgery/orthopedic practices. A paper-based survey consisting of 15 questions evaluated information regarding smartphone and medical app usage behavior. In addition, suggested app functions were rated using Likert scales. Descriptive statistics and binary log-binomial regression were performed.

**Results:**

A total of 1055 questionnaires were included in our statistical analysis. Approximately 89.57% (945/1055) of the patients in this study owned a smartphone. Smartphone ownership probability decreased with every decade of life and increased with higher levels of education. Medical information was obtained via mobile web access by 62.65% (661/1055) of the patients; this correlated with smartphone ownership in regard to age and educational level. Only 11.18% (118/1055) of the patients reported previous medical app usage, and 3.50% (37/1055) of the patients received an app recommendation from a physician. More than half (594/1055, 56.30%) of the patients were unwilling to pay for a medical app. The highest rated app functions were *information about medication*, *behavioral guidelines*, and *medical record archival*. An improved treatment experience was reported through the suggested app features by 71.18% (751/1055) of the patients.

**Conclusions:**

Mobile devices are a widely used source of information for medical content, but only a minority of the population reported previous medical app usage. The main target group for medical apps among patients receiving orthopedic and trauma surgical care tends to be the younger population, which results in a danger of excluding fringe groups, especially the older adults. Education seems to be one of the most important pull factors to use smartphones or a mobile web connection to obtain health information. Medical apps primarily focusing on an optimized patient education and flow of information seem to have the potential to support patients in health issues, at least in their subjective perception. For future target group–oriented app developments, further evidence on the clinical application, feasibility, and acceptance of app usage are necessary in order to avoid patient endangerment and to limit socioeconomic costs.

## Introduction

Today’s health care professionals are faced with patients who are increasingly adapted to digitalization by using smartphones as tools for communication and information or data collection in their daily private and professional lives [1]. Before the era of the World Wide Web, patients often had only limited access to medical literature and therefore were completely dependent on the expertise of medical professionals. Ubiquitous access to the internet has fundamentally changed the information behavior regarding general knowledge of (non) medical issues for the majority of people [2]. This development has been enhanced by the widespread use of mobile web connections via smartphones. Smartphone ownership in Germany has increased steadily in recent years consecutively. In the first quarter of 2018, 87% of internet users had used smartphones or mobile devices to go online. Smartphone ownership and the associated mobile web usage has also risen globally [3,4].

Mobile health (mHealth) tools such as medical apps can enable patients to play a more active role in their health care [5]. In the past, health care services and medical information were often bound to medical facilities. Nowadays, by using mobile devices, a large target group can be reached to improve patient monitoring and self-engagement [4]. This offers the opportunity to address users who are otherwise difficult to access regarding health topics, such as older adults, younger people, or those living in rural regions with a low level of medical infrastructure [6,7]. In the course of the rapid development in the field of medical apps, not only have patients benefitted from this technology but physicians have also fundamentally changed their information behavior by using smartphones and apps to access web-based medical resources during their clinical routine in recent years [8]. The use of smartphones and medical apps seems to be very popular among trauma surgeons and orthopedic surgeons as well. In Germany, the majority (79.1%) of trauma and orthopedic surgeons reported the use of smartphones and medical apps (64.4%) in their daily clinical routine [9]. Despite the extensive possibilities arising from the use of this evolving technology, the evidence base is currently still limited and there is a need for further studies [10,11]. In orthopedic and trauma surgical care, apps can be used, for example, for preventing injuries. App-based training is able to prevent sport injuries such as anterior crucial ligament sprains [12]. Patients with musculoskeletal pain such as chronic low back pain can also benefit from app support [13]. However, the integration and use of smartphones and medical apps in medical care, especially in the fields of orthopedic and trauma surgery, are still in an early developmental stage. Nevertheless, there seems to be numerous indications that mHealth solutions might have an additional benefit in the treatment of patients who have undergone orthopedic and trauma surgery. The exact requirements and level of acceptance of medical apps from a patient’s point of view are currently unknown. The target group for mHealth is also speculative and vaguely defined. Currently, the medical app features that are specifically important for these patients are still unknown. Therefore, it is essential to evaluate both the target group and patients’ requirements for future patient-oriented medical app developments in the field of orthopedic and trauma surgery.

## Methods

### Study Design and Execution

This prospective, multicenter study was conducted between August 2018 and December 2019. Paper questionnaires were distributed to patients in a level 1 trauma center in western Germany (Essen University Hospital) and in 3 private practices with a focus on outpatient care in trauma and orthopedic surgery during their outpatient treatment in the facility. The questionnaires were handed out to the patients by the assistant staff at the patient registration desk. After giving their consent to participate in the study, patients were requested to drop the completed questionnaire in a labeled container. Participation was anonymous and on a facultative basis. All investigations on humans were carried out with the consent of the responsible ethics committee in accordance with national law and in accordance with the Declaration of Helsinki of 1975 (current revised version). Inclusion criteria were as follows: (1) patients undergoing ambulatory treatment in the aforementioned institutions, (2) patients aged ≥15 years and ≤90 years, and (3) existing consent for study participation. Exclusion criteria were as follows: (1) patients aged <15 years or >90 years and (2) missing declaration of consent for participation in the study

### Survey Development

**Figure 1:**
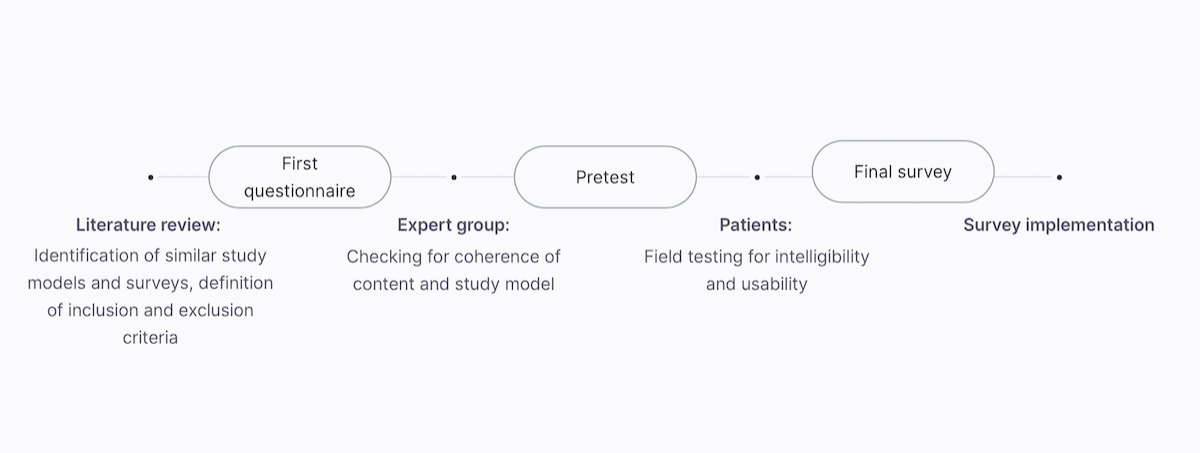
Schematic illustration of the survey development process.

As there is no gold standard for surveys in mHealth, a thorough literature review was conducted. App-related questions were developed and modified based on an already established survey [14]. The questionnaire was tested among a group of medical experts with know-how in the field of digitalism and survey development firstly and patients secondly. Based on feedback from the pretest survey, the final survey was created (Figure 1). A final questionnaire consisting of 15 questions was created. There were 3 sections in the survey. First, patients were asked about demographic characteristics (sex, age, educational background, insurance status, and type of treatment). The second part evaluated behavioral information regarding the patient’s use of smartphones and medical apps. Additionally, patients were asked whether they had a smartphone, a mobile web connection, and if they used their internet access for medical research. In addition, it was evaluated if the study patients used medical apps and whether they had ever received a recommendation for a medical app by a physician. Next, they were asked about their willingness to pay for an app in a medical context. Finally, patients were asked to rate the 10 proposed features of a fictitious smartphone app on a 6-point Likert scale in order to determine their preferences for the app. Patients were also able to indicate whether they felt that they would benefit from these app features in terms of treatment experience.

### Statistical Analysis

The completed questionnaires were returned to the investigator and given an identification number, which allowed conclusions about the institution collecting the data. The data were then entered into a Microsoft Excel worksheet (Version 15.18, Microsoft Corporation) and transferred to SAS 9.4 (Cary) for statistical analysis. Descriptive statistics were calculated for all items. In our statistical analysis, we classified patients with a university degree or a university (German) entrance qualification (13 years of school education) as having the highest level of education. Patients who had attended school for 10 years and stated that they had the corresponding German school certificate (“mittlere Reife”) were considered to have an average educational level. If the patients stated that they had no school diploma or the lowest German school certificate (9 years in school, “Hauptschulabschluss”), they were defined as having a low level of education. In addition, ranking of the best-rated app function was generated by ordering the mean ratings per app function. Ratings were on a scale of 1 (very important) to 6 (very unimportant). Rank 1 was considered to be the best-rated app with a consecutively low score. Since the app functions were evaluated using ordinal Likert scales, the median was chosen as the comparative location parameter. Furthermore, the interquartile range was determined. We performed Pearson chi-square test to determine whether there was a statistically significant association between the variables. All prevalence ratios and 95% confidence intervals were age-adjusted and sex-adjusted and were derived from log-binomial regression models.

## Results

### Pretest

Adjustments regarding content coherence, redundancy, and layout were made based on feedback from the pretest. Particular attention was paid to achieve only a short processing time for the survey in order to keep the response rate as high as possible. The patients took an average of 80 seconds to complete the survey.

### Descriptive Statistics and Demographic Data

A total of 1331 questionnaires were distributed between August 2018 and December 2019. Of these, 1132 (85.05% response rate) were completed and returned. Seventy-seven patients did not meet the inclusion criteria. Therefore, 1055 questionnaires were included in the statistical evaluation. In the trauma center, 58.39% (616/1055) of the questionnaires were collected, while 41.61% (439/1055) of the questionnaires were obtained via the 3 private practices. The study consisted of 60.66% (640/1055) female patients and 39.34% (415/1055) male patients. The median age of the subjects was 45 years with an interquartile range of 30-59 years. Most patients were in the age group of 46-55 years (193/1055, 18.29%). Most of the patients had an average (314/1055, 29.76%) or high (511/1055, 48.44%) educational level; 19.24% (203/1055) of the patients reported a low educational status, while 2.56% (27/1055) of the patients were secondary school pupils. Most of the study patients (910/1055, 86.26%) had statutory health insurance, while 145 patients (13.74%) stated as having private health insurance. A total of 1015 (96.21%) patients were undergoing outpatient treatment, while 40 (3.79%) were being treated in an inpatient setting.

### Smartphone Usage Behavior

A large number of patients reported smartphone ownership (945/1055, 89.57%) and mobile web access (942/1055, 89.28%). A statistically significant correlation of smartphone ownership with higher educational level (odds ratio 1.13, 95% CI 5%-22%; *P*<.01) and decreasing age (odds ratio –1.03, 95% CI 1.6%-3.2%; *P*<.01) could be proved. More than half of the patients (661/1055, 62.65%) stated that they used their mobile web access to obtain medical information, while 394 (37.35%) patients did not use their mobile web access for this purpose. Almost half (164/334, 49.1%) of the older patients (≥56 years) reported that they searched for health information online compared to 69.3% (251/362) of the patients younger than 36 years. A significant link between using a mobile web connection for medical research and age was seen in the study cohort. With every increased decade of age, the probability of using mobile internet access for medical information decreased by 6% (95% CI 4%-8%, *P*<.01) relatively. Furthermore, there was a correlation between higher level of education and the use of mobile web access for medical information. The probability of patients with average and high level of education to use mobile web access to obtain medical information was 40% (95% CI 16%-69%, *P*<.001) and 70% (95% CI 43%-103%, *P*<.001) higher, respectively, than of those with a low educational level. Only a few patients (118/1055, 11.18%) reported that they already use apps in a medical context, while 88.82% (937/1055) of the patients stated that they had not used medical apps yet. Again, the significant influence of age was obvious; with every 10-year age increase, the probability that patients used medical apps decreased by 16% (95% CI 8%-24%, *P*<.001) relatively. In addition, this study revealed a connection between educational level and the use of medical apps. The probability for medical app usage among patients with an average or high educational level was 92% (95% CI 0%-268%) or 116% (95% CI 16%-300%), respectively, greater than that among patients with a low educational level. Furthermore, we found a statistically significant association between medical app usage or web-based obtainment of medical information and the overall feeling of treatment improvement through the use of the app features we offered (*P*<.01). Only a few patients had previously received app recommendations from a physician (37/1055, 3.51%), while 96.49% (1018/1055) of the patients had not received such recommendations (Table 1). When asked about the willingness to pay for a medical app, 56.30% (594/1055) of the patients were unwilling to pay money for medical apps, 10.71% (113/1055) of the patients were willing to pay up to €0.5 (US$ 0.54), and 23.60% (249/1055) of the patients would spend up to €3 (US$ 3.26). Almost 6.92% (73/1055) of the patients were willing to pay €7.50 (US$ 8.16), followed by 2.46% (26/1055) of the patients who would pay up to €15 (US$ 16.31) for a health app. The median was €1 (US$ 1.08) with an interquartile range of €1-3 (US$ 1.08-3.25). We identified a statistically significant correlation between higher patient age and an increased willingness to pay (*P*<.01).

**Table 1:** Key data on smartphone and medical app usage behavior of the patients in this study (N=1055).

Usage behavior		Values, n (%)
**Smartphone ownership**		
	Yes	945 (89.57)
	No	110 (10.43)
**Mobile web access**		
	Yes	942 (89.28)
	No	113 (10.71)
**Is web access used to obtain medical information?**		
	Yes	661 (62.65)
	No	394 (37.35)
**Medical app use**		
	Yes	118 (11.18)
	No	937 (88.81)
**Has a medical app ever been recommended by a doctor?**		
	Yes	37 (3.51)
	No	1018 (96.49)

### App Functions

**Figure 2:**
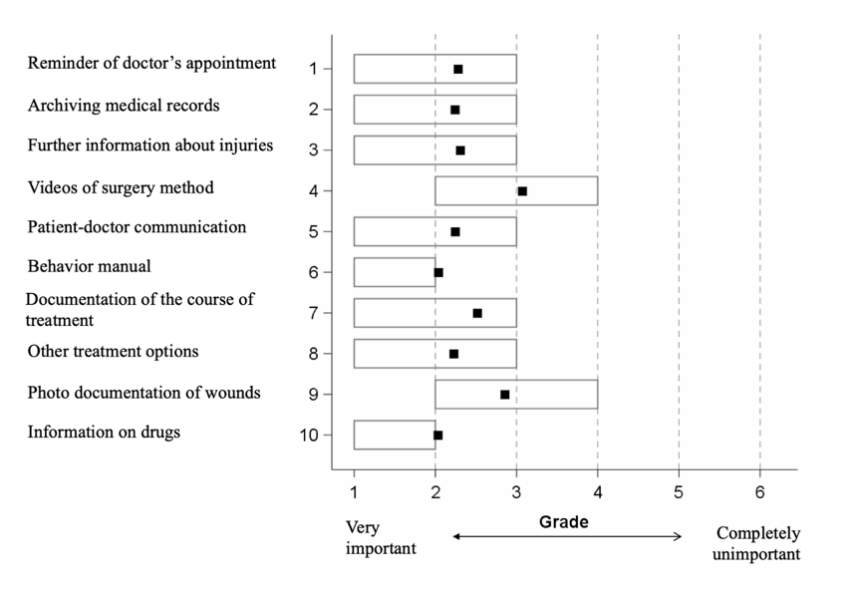
Evaluation of the features of a fictitious app by the patients in trauma surgical care (N=1055). The evaluation was based on a 6-point Likert scale from 1 (function very important) to 6 (function very unimportant). Boxes indicate interquartile ranges, and squares indicate median grades.

An app feature that provides a patient with information about the prescribed medication was rated best by patients in this survey (rank 1, score 1.91). An app providing behavior guidelines or discharge instructions following surgical procedures (eg, for traumas) was the second best-rated feature (rank 2, score 2.04). Archival of medical records was the third best-rated app function in this study (rank 3, score 2.19) (Figure 2). Across all age groups, 71.18% (751/1055) of the patients indicated that the aforementioned app features would improve their treatment experience, while 28.82% (304/1055) negated this. Age had a significant influence on a perceived treatment improvement as a result of these app features because the app’s functions were more likely to give younger patients a more positive feeling about their treatment. With every 10-year increase of age, the probability that patients would benefit from these app features decreased relatively by 4% (95% CI 2%-6%, *P*<.01). However, even the majority of the patients older than 65 years (110/163, 67.5%) stated that they felt more comfortable in their treatment experience as a result of the app’s features.

## Discussion

### Principal Findings

This study proved that smartphone usage and mobile web access are already widespread among patients receiving orthopedic and trauma surgical care. Mobile devices seem to be a widely used source of information for medical content. Education is one of the most important pull factors to use smartphones or a mobile web connection to obtain health information, which is in line with that reported in previous research [15-17]. Only a minority of the population stated the previous use of medical apps. Although many physicians use apps in their daily clinical routine, seemingly, not many consider medical apps to be important tools for patient care [8,9,18]. Consequently, very few patients reported having received a physician’s recommendation for a medical app. Patients who reported that they had already used medical apps tended to be younger and had a higher educational level, which concurs with the findings of prior investigations [16,19]. As more than half of the patients were unwilling to pay for a medical app, a high sensitivity to the price of apps in a medical context might be apparent, which is consistent with that reported in previous studies [20,21]. More than 70% of the patients, including older adults, felt an additional benefit from the features suggested in the fictitious app presented. It is not surprising that younger people have a slightly greater app affinity as app users tend to be younger in general. Target group–specific apps, therefore, seem to have the potential to support patients in health issues, at least in their subjective perception [22]. The most demanded app functions focused primarily on an optimized patient education and flow of information. *Information on drugs* was the highest rated app specification and might play an important role in pain management and self-management and therefore minimize drug-related complications [23,24]. A large number of medication management apps already exist, but none that is explicitly targeted at patients in a surgical specialty, where pain killers, especially opioids, are frequently prescribed [25-27]. *Posttraumatic and postoperative behavioral guidelines or discharge instructions* were also highly important (second highest rated). Former studies have already shown the prevalence of noncomprehensive discharge instructions, particularly among older adults who represent the major proportion of the trauma surgery patient clientele [28,29]. Important behavioral guidelines or other information may not be conveyed to the patient in a comprehensible manner [30]. In these instances, apps with implemented behavioral guidelines might support the patient in a postinterventional setting in addition to the conventional treatment [31].

### Limitations

This study has some limitations. This study was conducted solely in the field of orthopedics and trauma surgery in the German health care system. Because of this limited scope of application, the gained evidence is only valid for the respective target group. This necessitates the need to explore any additional requirements that may be needed for medical apps in emerging nations or more rural nations and health care systems. Moreover, it is crucial to evaluate whether the requirements of patients for mHealth apps vary in disciplines other than orthopedic and trauma surgery. The results of this study are in line with the patient’s requirements for apps in the field of chronic diseases of the musculoskeletal system. Using medical apps in rheumatology seems to be beneficial for the patient’s outcome. However, the usage of mHealth among patients with rheumatism is very limited and eHealth literacy is rather poor too [32]. A paper-pencil–based survey was conducted to address all age groups equally, as older patients may not respond to a web-based survey. Future studies may choose a web-based survey to expand the number of patients. In addition, a new nonvalidated questionnaire was created for this study, which has not yet been used in large clinical trials. However, due to the pretest, disadvantages could be omitted. Since the study patients rated only 10 different suggested app features, it remains unclear whether other features might be of higher importance. Prospective app development must evaluate the importance of additional features.

### Outlook

Medical apps are a milestone in patient care and doctor-patient interaction. These apps are rapidly moving into health care and through further developments, might offer a wide range of possibilities for patients undergoing trauma surgery in the future. The patient structure in the field of orthopedic and trauma surgery is heterogeneous and is partly dominated by the older adults. One great challenge in the development of medical apps—also from an ethical point of view—is to find ways to address all patients equally. Future app developments should take target group–specific app requirements into serious consideration. As the best rated app features result in an optimized information flow, a remarkable information deficit or knowledge deficit during and after medical treatment might be inferred. Apps or other mHealth-based solutions may offer the opportunity to compensate for such deficiency. Information can literally be made available at the patients’ fingertips and would be accessible at any time. It is questionable whether the majority of the patients reported a possible benefit from an app-supported treatment, as only a very small minority had ever received an mHealth app recommendation from a physician. After the initial ground-breaking steps, the German legislature recently gained considerable momentum in the direction of a stringent national digitization strategy. The “Law for better care through digitization and innovation” (Digitale-Versorgung-Gesetz) passed by the Bundestag on November 7, 2019 paved the way for the prescription of apps, the improved use of web-based video consultation services, and greater data security in the communication of health data. This highlights the necessity to broaden the acceptance of this technology among treating physicians [33,34]. Given these possibilities, it will be necessary to gain well-founded evidence for the effectiveness of this technology in order to prevent high socioeconomic costs for inadequate apps and those that may endanger patients in Germany. Requirements such as data security and interoperability with clinical information systems will be mandatory to establish this technology in clinical routine and to increase its acceptance. As only a few patients in this study reported previous medical app usage, satisfactory or affordable offers seem to be lacking. Surgeons strive to support their patients by finding appropriate apps that address their specific needs. High-quality apps must be identified by involving physicians from corresponding specialties and professional associations [35]. Reimbursement programs might be helpful to broaden the use of apps, as our findings demonstrated a high price sensitivity. Medical professionals will be responsible to ensure that medical apps are not solely economically driven and have the primarily goal of improving patient health care. Future app developments should be based on medical guidelines and be accompanied by the expertise of medical professionals in order to create more transparency and benefit for patients.

### Conclusions

Mobile devices are a widely used source of information for medical content, but only a minority of the population reported previous medical app usage. The main target group for medical apps in orthopedic and trauma surgery tends to be the younger population, which results in a danger of excluding fringe groups, especially the older adults. Education seems to be one of the most important pull factors to use smartphones or a mobile web connection to obtain health information. Therefore, medical apps primarily focusing on an optimized patient education and flow of information seem to have the potential to support patients in health issues, at least in their subjective perception. For future target group–oriented app developments, further evidences on the clinical application, feasibility, and acceptance of app usage are necessary in order to avoid patient endangerment and limit socioeconomic costs.

## Abbreviations

mHealth: mobile health
